# Comparative Study of the Collective Dynamics of Proteins and Inorganic Nanoparticles

**DOI:** 10.1038/srep41671

**Published:** 2017-02-08

**Authors:** Esmael J. Haddadian, Hao Zhang, Karl F. Freed, Jack F. Douglas

**Affiliations:** 1 Biological Sciences Collegiate Division, University of Chicago, Chicago, IL 60637, USA; 2 Department of Chemical and Materials Engineering, University of Alberta, Alberta, T6G 1H9 Canada; 3 Department of Chemistry, James Franck Institute, and Computation Institute, University of Chicago, Chicago, IL 60637, USA; 4 Materials Science and Engineering Division, Materials Measurement Laboratory, National Institute of Standards and Technology, Gaithersburg, Maryland 20899, USA

## Abstract

Molecular dynamics simulations of ubiquitin in water/glycerol solutions are used to test the suggestion by Karplus and coworkers that proteins in their biologically active state should exhibit a dynamics similar to ‘surface-melted’ inorganic nanoparticles (NPs). Motivated by recent studies indicating that surface-melted inorganic NPs are in a ‘glassy’ state that is an intermediate dynamical state between a solid and liquid, we probe the validity and significance of this proposed analogy. In particular, atomistic simulations of ubiquitin in solution based on CHARMM36 force field and pre-melted Ni NPs (Voter-Chen Embedded Atom Method potential) indicate a common dynamic heterogeneity, along with other features of glass-forming (GF) liquids such as collective atomic motion in the form of string-like atomic displacements, potential energy fluctuations and particle displacements with long range correlations (‘colored’ or ‘pink’ noise), and particle displacement events having a power law scaling in magnitude, as found in earthquakes. On the other hand, we find the dynamics of ubiquitin to be even more like a polycrystalline material in which the α-helix and β-sheet regions of the protein are similar to crystal grains so that the string-like collective atomic motion is concentrated in regions between the α-helix and β-sheet domains.

Science thrives on analogy. A phenomenon that occurs in one field of science is found to arise in another where the time and spatial scales and the materials involved can be vastly different, yet the essential physical phenomenon and corresponding mathematical description strikingly apply to both. Karplus and coworkers^1^ have argued for such an analogy between the dynamics of proteins in their biologically active folded ‘native’ state and inorganic nanoparticles (NPs) in their ‘pre-melted’ state. While it is generally recognized that these ‘particles’ have similar dimensions, typically on the order of a few nanometers, it is a natural to believe that inorganic NPs and proteins display rather different structure and dynamics. This difference in perception is probably rooted in textbook images of NPs showing highly rigid structures with faceted surfaces that reflect their habit of ordering crystallographically. However, both experimental^2^,^3^ and computational^4^,^5^ examinations of the structure of NPs at elevated temperatures (*T*) reveal that metallic NPs can exhibit a much more disordered structure and complex dynamics than anticipated from textbook cartoons of these particles. The small size of NPs and their relatively large surface area normally imply a significant downward shift of the melting temperature of the NPs^5^,^6^,^7^,^8^,^9^,^10^,^11^,^12^ and appreciable excitation of motion on the surfaces of these particles at *T* well below the melting point temperature, *T*_*m*_, of the bulk material^4^. Even at *T* as much as 30% to 50% below the NP *T*_*m*_, these nano-crystals exhibit a relatively high interfacial mobility, although it is simplistic to characterize their interfacial dynamics as being like a ‘liquid’^13^,^14^,^15^,^16^. This ‘pre-melted’^5^,^12^,^17^ condition makes the interfacial regions of metal NPs highly mobile^13^, and significant fluctuations in particle shape can emerge when the NPs actually have a size comparable to 1 nm^18^. (We consider the transition to a ‘pre-melted’ surface state to be a dynamical rather than a structural transition in which the interfacial region of a crystal is transformed into a state with high atomic mobility. The dynamics in this layer then more closely resemble those of a superheated crystal than a simple liquid, as envisioned by models of surface melting). In fact, the dynamics in the interfacial regions of these NPs is strikingly similar to the dynamics of glass-forming (GF) liquids, a phenomenon that is elaborated below in comparisons to our ubiquitin simulations^4^. Our findings below broadly support the suggestion by Karplus and coworkers^1^ that folded proteins have some features similar to ‘pre-melted’ inorganic NPs, a suggestion offering a potentially new paradigm for understanding the biological activity of proteins. Reversing the analogy, these observations might aid in our understanding of the catalytic behavior of small NPs of normally chemically inert substances such as Au^19^,^20^,^21^.

Our recent simulations of the interfacial mobility and collective dynamics of crystalline Ni nanoparticles at elevated *T* have revealed significant “dynamic heterogeneity” in the interfacial dynamics of these particles. In particular, the atomic dynamics of these NPs exhibit colored noise (explained in part D of the results section) in the fluctuations in potential energy and in the amplitude of average particle displacement on a *ps* timescale^13^. This type of heterogeneous dynamics is also characteristic of grain boundaries^22^ at elevated *T*, and the same type of collective motion has been established in simulations of both metallic^14^,^23^,^24^ and polymeric GF liquids^25^ and in the dynamics of lipid membranes^26^,^27^. We emphasize that the chemical heterogeneity of the polymer, polymer length and even chain connectivity is not of significance for the observation of this type of dynamic heterogeneity, although these molecular variables do influence the extent of this motion at a given *T*. Dynamic heterogeneity is evidently a universal property of condensed disordered matter exhibiting strong interparticle interactions. We may then anticipate this to be a general feature of the dynamics of globular proteins in solution at low *T* or concentrated molecular environment found within cells.

The protein-inorganic NP analogy suggests that quantitative methods for characterizing the collective motion in GF liquids^4^,^22^,^28^ could be adapted to describe collective motion in proteins (This methodology was recently applied to analyze the collective dynamics found in lipid membranes^26^). As expected, we find collective motion within ubiquitin that takes the form of string-like atomic exchange displacements. We also see other general features of the dynamics of glass-forming liquids in association with this collective motion- colored noise in potential energy and particle displacement fluctuations, and quake-like particle displacement events. These observations remarkably accord with the surface-melted NP picture of proteins suggested by Karplus and coworkers^1^.

In addition to observing string-like collective motion within ubiquitin, we find that the scale of this collective motion in glycerol solutions becomes greatly attenuated by the addition of water, an effect similar to raising the temperature of the solvent. This trend is reminiscent of dynamic neutron scattering observations indicating a reduction of correlated particle exchange motions in proteins upon passing from being folded to unfolded upon heating^29^. We also observe a power law scaling in the intensity of the particle displacements for particles undergoing collective atomic exchange motion, where the power in this relation depends on solvent composition. Such intermittent motion has been observed before in Ni NPs^4^,^13^,^30^ and on a rather different scale in the phenomenology of earthquakes^31^,^32^.

Some of our observations on the dynamics of ubiquitin at first seemed to deviate significantly from expectations based on the analogy between the dynamics of surface-melted NPs and proteins suggested by Karplus and coworkers^1^. While our simulations confirm their suggestion that the cores of folded proteins, such as ubiquitin, are relatively dense, which is rather generally for Ni and other inorganic NPs, the amplitude of atomic motions within ubiquitin *does not* track these density changes. In particular, both the amplitude of atomic motion, as measured by the average Debye-Waller factor 〈*u*^2^〉 and the radially averaged density of the protein about its center of mass are *large* near the protein’s center, a feature that no doubt facilitates large-scale protein conformational rearrangement in this protein. However, this unexpected finding is not really in contradiction with the dynamics of inorganic nanoparticles as small as ubiquitin. For inorganic NPs having a diameter as small as 1 nm, a relatively large 〈*u*^*2*^〉 near the NP center has been found to arise from the central atoms being constantly pushed out from the NP center by large scale collective particle motions that ‘short-circuit’ through the center of the NP, initiating large fluctuations in the NP shape because of the disruptive nature of these events on atomic packing^33^. In larger NPs, the collective motion remains localized to the interfacial region of the NP. We observe a similar lack of correlation between the variations of density and mobility which has been observed recently in GF polymer films^34^. So even if the protein-surface-melted NP analogy remains intact, we have the important lesson that naïve ‘free volume’ arguments relating local mobility to local density can fail miserably at a molecular scale.

Prompted by reviewer comments, we examined some proteins larger than ubiquitin, and found that the maximum in 〈*u*^*2*^〉 near the protein center no longer exists in the larger folded protein, bovine serum albumin. Therefore, even the mobile core effect of ubiquitin accords with the surface-melted NP model of proteins when the size effects are considered in both inorganic and organic nanoparticles. At the same time, the mobile core of ubiquitin is probably not a typical property of proteins; these macromolecules have special biological functions and structures so that no universal trend between the interior mobility and density of proteins exists.

## Results

Before discussing our simulation results for ubiquitin in water and glycerol, a few words about the intermolecular potentials utilized in our work are required for understanding the findings below. We utilize the most recent variant of CHARMM (version 36) family of force fields^35^ and in Supplementary Material we discuss basic aspects of the dynamics of water and glycerol that derive from this family of potentials. Although this force field is widely utilized for the simulation of biological macromolecules, we realized after the completion of our simulations that the CHARMM36 force field should be utilized to study protein dynamics with caution. The rather serious shortcomings of this family of potentials can be appreciated from the fact that the modified TIP3P water model^36^, which is central to CHARMM, has an equilibrium melting temperature *T*_*m*_ near 146 K^37^ rather than the melting temperature of real water, 273 K. This aspect of the TIP3P water model can be expected to significantly influence all aspects of protein dynamics in which the collective dynamics of water is important. Correspondingly, we find that ubiquitin in TIP3P water model exhibits rather little collective dynamics at room temperature. In Supplementary Material, we demonstrate that this model of water by itself exhibits *essentially no dynamic heterogeneity* at either room temperature (300 K) or at temperatures much below the melting temperature of real water (e.g., *T* = 250 K) and contrast this behavior with SPC/E water^38^,^39^, which has a more physically reasonable^37^ melting temperature, *T*_*m*_ (SPC/E) = 215 K. These disturbing findings for TIP3P water model are contrasted with estimates of dynamic heterogeneity in SPC/E water (whose equilibrium melting temperature is a more physically reasonable^37^ 240 K). In this water model, there is appreciable collective dynamics near room temperature that follows the pattern found broadly in glass-forming liquids^40^,^41^,^42^. Fortunately, the glycerol model utilized by CHARMM provides a good example of a model dynamically heterogeneous solvent (See Jahn *et al*.^43^ and Simulation Methods), so we can explore the coupling of this dynamically heterogeneous solvent to the dynamics of ubiquitin. We find below that this solvent-protein pair is indeed suitable for this purpose. With the advantage of hindsight, we now view the unphysical dynamical nature of TIP3P water as being useful for our discussion provided we consider this water model to be a hypothetical liquid devoid of collective molecular motion at all *T* relevant to real liquid water. CHARMM36 glycerol, on the other hand, should be closer to the dynamical character of real water near room temperature because of its dynamically heterogeneous nature over wide range of *T*. With these caveats in mind regarding our usage CHARMM force field, we now describe our findings from the simulations of ubiquitin where we have adapted liquid state tools developed for glass-forming liquids to quantify the collective dynamics within ubiquitin dispersed in water, glycerol and a water-glycerol mixture to tune the extent of collective motion within the protein.

### Van Hove Correlation Function *G*_*s*_(*r, t*)

Calculations of the van Hove correlation function provide a first check regarding whether the dynamics of ubiquitin are similar to those of GF liquids in exhibiting features relating to the phenomenon of ‘dynamic heterogeneity’. The self-part of the van Hove function *G*_*s*_(*r, t*) describes the probability distribution that an atom becomes displaced from its initial position to a distance *r* after a time interval, Δ*t*. Mathematically, the self-part of the van Hove correlation function *G*_*s*_(*r, t*) is defined as^44^,^45^,^46^,


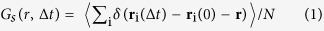


where **r**_*i*_ designates the atomic position of the *i*^th^ particle, **r** is a general position in space having a distance *r* = |**r**| from the origin, and *N* is the total number of atoms. The Fourier transform of this quantity, the self-intermediate scattering function *F*_s_ (**q**, *t*) with **q** the scattering ‘wavevector’, is accessible experimentally from incoherent quasi-elastic neutron scattering measurements^47^. The van Hove function for an ideal fluid in which the particles undergo Brownian motion reduces to a Gaussian function *G*_*s*_^*o*^(*r, t*),


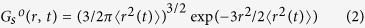


where the mean square average atomic displacement 〈*r*^2^(Δ*t*)〉 is p*r*oportional to Δ*t*. This same functional form also arises for harmonically localized atoms at long times where 〈*r*^2^(Δ*t*)〉 becomes independent and cha*r*acterizes the scale of particle localization and is generally observed at rather short times where particle motion is inertial in nature. A direct comparison of *G*_*s*_(*r, t*) to *G*_*s*_^*o*^(*r, t*) permits a quantification of the extent of non-Gaussian behavior, but, in practice, it is simpler to use the moments of *G*_*s*_(*r, t*) to characterize deviations from Gaussian particle displacement dynamics. In particular, it is conventional to define the “non-Gaussian” parameter *α*_2_(Δ*t*) as a ratio of the second and fourth moments of distribution of *G*_*s*_(*r, t*),


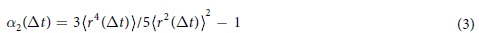


during the time interval Δ*t*. By definition, this quantity equals 0 for a Gaussian process. For very small Δ*t*, shorter than the times requited for particle collisions, inertial particle motions dominate and displacements are Gaussian so that *α*_2_ is nearly zero, while at very large times Δ*t* particles in a fluid execute a random walk type of motion so that *α*_2_ ultimately becomes small again. Outside these limiting long and short time regimes, the magnitude of *α*_2_(Δ*t*) provides both a measure of the extent to which particle displacements are Gaussian, as described above, and the magnitude of mobility fluctuations (i.e., “dynamic heterogeneity”)^28^.

**Figure 1:**
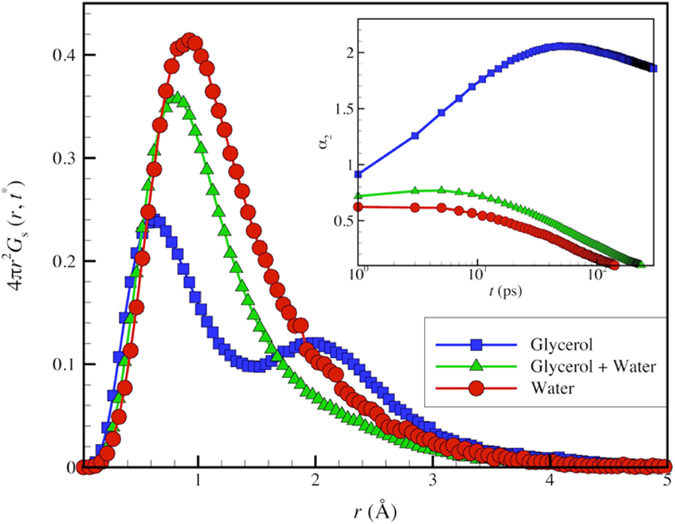
van Hove self-correlation function *G*_*s*_(*r*, Δ*t*) of ubiquitin in glycerol/water solutions. The inset shows the non-Gaussian parameter, *α*_*2*_ of ubiquitin where all protein atoms are included in the analysis. The peak in *α*_*2*_ and a secondary ‘hopping peak’ in *G*_*s*_(*r*, Δ*t*) observed for glycerol are signatures of dynamic heterogeneity in the dynamics of GF liquids. The addition of water suppresses these features due to the uncooperative nature of TIP3P water model.

The inset of Fig. 1 displays *α*_2_(Δ*t*) of all protein atoms as a function of Δ*t* for ubiquitin in glycerol, TIP3P water, and in a mixture of glycerol-TIP3P solution. Evidently, there is a time t* for glycerol at which *α*_2_(Δ*t*) exhibits a peak. This characteristic timescale is a universal feature of GF liquids^24^,^48^, along with a rapidly growing structural relaxation time *τ* upon cooling. In contrast, we observe no peak in *α*_2_(Δ*t*) at a finite time in our ubiquitin-water simulations. This absence of collective motion can be traced back to the TIP3P water, which likewise exhibits no peak in *α*_2_(Δ*t*) at a finite time over a wide *T* range below room temperature (See Supplementary Material). Evidently, TIP3P is a remarkably homogeneous solvent from a dynamical standpoint and this property is imparted to the protein by virtue of a strong coupling between the dynamics of the protein to the solvent (See discussion in Supplementary Material). There is no point then in studying the dynamics of ubiquitin at lower temperatures based on CHARMM force field simulations.

Figure 1 displays the van Hove correlations function *G*_*s*_(*r*) evaluated at the characteristic time t* at which the dynamical heterogeneity of the solvent is maximally exhibited. The multiple peaks in *G*_*s*_(*r*) seen for ubiquitin in glycerol are characteristic of ‘hopping’ motions of atoms to preferentially “quantized” distances. This common feature of the dynamics of GF liquids^22^,^23^,^28^ is entirely suppressed at room temperature when TIP3P water is added. Comparison between simulation estimates of diffusion coefficient (*D*) from different molecular potentials to experiment^14^ are best made at common reduced temperature, *T*/*T*_*m*_, and we can then directly understand the problems with TIP3P water model in relation to studying the dynamics of protein solutions. TIP3P has a melting temperature of *T*_*m*_ = 146 K rather than 273 K so that the reduced temperature *T*/*T*_*m*_ for TIP3P is high for almost any simulation temperature relevant to real water in its liquid state. At first, we did not appreciate this aspect of TIP3P water model and we wasted computational and analysis time through a progressively lowering of *T* in our simulations, only to find that there was essentially no change in the extent of collective motion in either the protein or the water molecules as temperature was lowered below room temperature. This aspect of TIP3P water allows us to understand why the3re is no hopping peak in the van Hove function or peak in *α*_2_(Δ*t*) in Fig. 1. We suggest TIP3P water should be thought of as an ideal solvent devoid of collective motion that serves as a useful reference point for solvents that exhibit heterogeneous dynamics near room temperature, such as real water and glycerol. Below we describe the impact of dynamic heterogeneity suppression in TIP3P water on other aspects of ubiquitin dynamics.

### 〈*u* ^2^〉-A High Frequency Measure of Protein ‘Softness’ and Local Mobility

**Figure 2:**
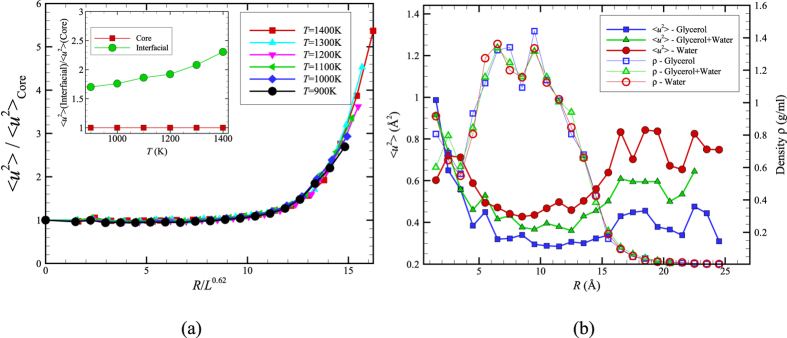
Radially averaged Debye-Waller factor 〈*u*^2^〉 for a Ni NP versus the atoms of ubiquitin. (**a**) The 〈*u*^2^〉 values for the Ni NP atoms with *R* = 2 nm (whose interatomic interactions are modeled by the Voter-Chen Embedded Atom Method (EAM) potential^53^) are normalized by their value at the center of the NP and *R* is the radial distance from the NP center^13^. Inset compares 〈*u*^2^〉 values in the NP interfacial region to values in the NP core. All our radially averaged 〈*u*^2^〉 data can be described as universal function of *R*/*L*^*0.62*^ where *L* is the string length (defined in text). The width of the interfacial region of enhanced mobility near the NP surface is apparently governed by the scale of collective exchange motion within the NP^13^. Recent work has demonstrated that *L* also governs the width of both the polymer-air interfacial layer of a glass-forming polymeric liquid film^34^ and the interfacial layer of bulk crystalline Ni^14^ so that the existence of a mobile interfacial near the ‘softening temperature’ of the material seems to be a general property of condensed materials broadly. (**b**) The radial averaged 〈*u*^2^〉 of ubiquitin atoms in glycerol, water, and a glycerol-water mixture at *T* = 300 K where *R* is the radial distance from the protein center of mass. The addition of TIP3P water to the glycerol solution clearly ‘plasticizes’ the ubiquitin dynamics, i.e., increases the amplitude of local atomic displacements within the protein where this effect is greater near the protein periphery than the protein core. We also show the radially averaged local density *ρ* within the protein to emphasize that the local density *ρ* and the local ‘mobility’ 〈*u*^2^〉 do not exhibit similar trends with *R* in this protein. We show in Fig. 3 that this is not a universal behavior in the dynamics of proteins.

Karplus and coworkers^1^ also emphasized that the Debye-Waller factor 〈*u*^2^〉, the mean square displacement of the alpha carbon atoms after a caging time on the order of ps, should provide a good measure of local mobility within the protein. Zaccai and coworkers^49^ have promoted this quantity as a measure of protein ‘resilience’ or ‘softness’. Comparison of the radially averaged 〈*u*^2^〉 for ubiquitin and for a surface-melted nanoparticle allows assessing another implication about the physical nature of proteins versus that of inorganic nanoparticles. Figure 2(a) presents the radial average of 〈*u*^2^〉 for a 4 *nm* diameter Ni NP below its melting temperature (*T*_m_ = 1500 K) but in its ‘surface-melted’ state^13^. The mean amplitude of thermal motion exhibited in the fast dynamics of the Ni atoms is smaller in the core of the Ni nanoparticle and much higher in amplitude near the NP surface. Is the same trend observed in ubiquitin and other folded proteins?

Comparing the results for Ni NPs with similar radially averaged 〈*u*^2^〉 plots for ubiquitin (all atoms of the protein are considered, including the H-atoms) exposes a significant difference between the dynamics of inorganic NPs with *R* = 2 *nm* and ubiquitin (radius of gyration ≈ 1 *nm*). We see in Fig. 2(b) that the radially averaged 〈*u*^2^〉 for ubiquitin is relatively large in the *center* of the protein, regardless of the solvent composition. Lindorff-Larsen^50^ have experimentally observed a high mobility in the core of ubiquitin on a *ps* timescale and suggest that this is a common property of many proteins, which should have many ramifications for biological function. Hong *et al*.^51^ suggest that this behavior arises from the excitation of greatly enhanced local amplitude motions of the protein interfacial atoms due to their strong hydrogen binding interactions with the solvent and the indirect effect of these large anharmonic motions on atoms deep within the protein core through some type of unspecified collective motion linking the interfacial and protein core atoms^51^. The ‘softness’ of the protein core was inferred even earlier by Gekko and Hasegawa^52^, who examined the compressibility of 11 globular proteins in water where most of the proteins examined were reported to have a large internal compressibility. They further found that this unexpectedly high compressibility correlated strongly with protein stability and protein core hydrophobicity.

While Fig. 2(b) indicates the trend stated by Karplus and coworkers, i.e., the density in the protein interior (simply the average protein mass divided by the volume in a shell of radius *R*) is relatively high, similar to a dense fluid or crystal. However, this relatively high density apparently *does not* translate into the large reduction of mobility in the protein core that would be expected from naïve ‘free volume’ reasoning. Recent simulation modeling has repeatedly shown that free volume arguments are generally unreliable when applied locally^54^,^55^,^56^. Next, we consider a possible explanation for this unexpectedly high mobility in the ubiquitin core, an effect also observed in certain inorganic NPs.

How typical are these findings for ubiquitin? After all, ubiquitin is a rather small protein (radius of gyration ≈ 1 nm) and a maximum value of 〈*u*^*2*^〉 in their center. Inorganic NPs have also been observed in small atomic clusters having a similar size^33^. In particular, we have also investigated ‘small’ Ni NPs^18^ having a diameter ≈ 1 nm and found that these NPs exhibited a *qualitatively different* dynamics from the NP shown in Fig. 1. In particular, Yang *et al*.^18^ found that the atoms in the core of these smaller NPs are intermittently driven to the NP surface though a kind of collective motion within the NP, an effect that accounts for both the relatively large 〈*u*^*2*^〉 in the particle core and associated large scale NP shape fluctuations derived from these collective disturbances of the NP structure. In contrast, this effect *disappears* in larger NPs having a diameter of only a few nm since the collective motions within the particle remain localized to the NP interfacial region^4^.

**Figure 3:**
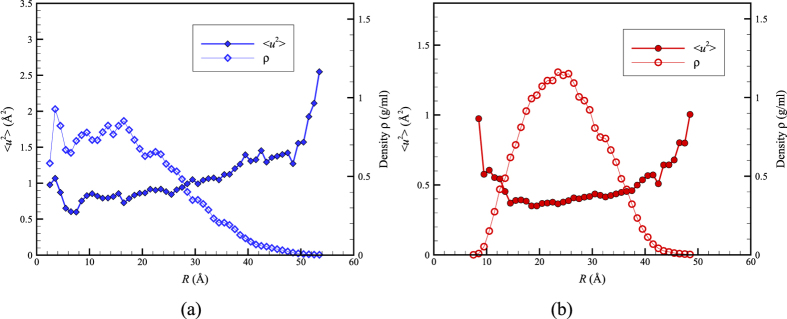
Radial averages of 〈*u*^2^〉 and density for bovine serum albumin (BSA, PDB id 4F5S) and insulin degrading enzyme (PDB id 4IOF) in water (TIP3P) at *T* = 300 K where *R* is the distance from the protein center of mass. (**a**) We see that *ρ* is relatively high, and correspondingly 〈*u*^*2*^〉 is relatively low, near the center of the relatively large globular protein BSA, a trend that is qualitatively similar to our Ni NP observations in Fig. 2(a) and that epitomizes the arguments of Karplus and coworkers. (**b**) The example of insulin degrading enzyme reminds us that proteins have special biological functions and structures so that no universal trend between the interior mobility and density of proteins exists. This protein has a binding cavity in its core that accounts for the low density in the center of the protein.

In order to clarify whether ubiquitin is behaving instead like an ultra-small inorganic nanoparticle and to address reviewer queries about the generality of our findings of a mobility maximum in the interior of ubiquitin, we then simulated two proteins larger than ubiquitin, bovine serum albumin (PDB id 4F5S; 583 residues) and insulin degrading enzyme (IDE, PDB id 4IOF; 990 residues), to determine if these proteins are more like the Ni NP data in Fig. 2(a). In Fig. 3(a), we illustrate the gradient in local dynamics and density within Bovine serum albumin (BSA), a relatively large ‘garden variety’ globular protein. In contrast to ubiquitin, this protein exhibits a variation in density ρ and 〈*u*^*2*^〉 that is qualitatively similar to the Ni NP in Fig. 2(b). In particular, the core of this protein has a relatively high density, while the local mobility in the core is relatively low, exactly as argued by Karplus and coworkers. On the other hand, an examination of the very large IDE protein (radius of gyration ≈ 2.5 *nm*) reveals that this pattern of behavior is not universal in larger proteins. We see in Fig. 3(b) that IDE has a relatively large mobility at its periphery, as found for all NPs considered, but this protein also has a relatively large mobility in its core as found for ubiquitin. In this example, this property is readily understandable since this protein has a large cavity in its core at which insulin binds and then becomes degraded. We may conclude that variability in the internal dynamics of proteins can then be expected given their diverse biological shapes and functions. Indeed, Henzler-Wildman and Kern have recently discussed how proteins acquire “dynamic personalities”^57^ because of the presence of cavities or other structural constraints that modulate their local dynamics.

The observations in Fig. 2(b) also provide insight into how glycerol influences the dynamics of proteins. In particular, notice that the radially averaged value of 〈*u*^*2*^〉 over the entire ubiquitin molecule is appreciably increased from 〈*u*^*2*^〉_rad aver_ = 0.41 in pure glycerol to 0.67 in pure water. Since 〈*u*^*2*^〉 is a measure of the local molecular ‘softness’^49^, this means that glycerol has an overall stiffening effect on the protein molecule. This stiffening effect of glycerol on protein dynamics is well recognized from previous experimental studies on proteins in mixed glycerol-water solutions^58^, which makes glycerol a useful ‘stabilizing’ agent for protein crystallization^58^ and as an molecular additive for enhanced protein preservation^59^.

Figure 2(b) also reveals that changes in 〈*u*^2^〉 accompanying the addition of water occur non-uniformly within the molecule. In particular, the enhancement of molecular motion is much greater near the protein core than at the protein-water boundary. The analogy of proteins with inorganic NPs greatly aids in understanding this initially puzzling trend. In addition to water general speeding up the dynamics of the protein by altering the relaxation dynamics of the solvent mixture (see Supplementary Information), the addition of water to glycerol can also be expected to alter the *thermodynamic stability* of the folded state of ubiquitin. A continuous upward shift of the denaturation temperature is normally found in proteins with increasing water content^60^,^61^,^62^, an effect rather similar to adding a denaturant, such as DMSO or urea, to an aqueous protein solution. The stabilizing effect of glycerol and various sugars has great significance for protein preservation^59^. We checked the possibility that the water was destabilizing the folded state of ubiquitin in comparison to glycerol by examining the distribution of conformational states, as quantified by the calculated protein radius of gyration *R*_*g*_ and hydrodynamic radius *R*_*h*_ for a large ensemble of protein structures from our MD trajectories (unpublished work). Consistent with our expectation that water makes the folded state of ubiquitin less stable than in glycerol, we find that the distributions of these basic measures of protein size are much broader for aqueous ubiquitin solutions. Moreover, our simulations indicate a dynamic coexistence between two bands of relatively open and closed ubiquitin conformational sates having significantly different average protein sizes, while these distributions were mono-modal and relatively narrow for ubiquitin in glycerol. This type of dynamic coexistence phenomenon is typical of “small” nanoparticles (diameter ≈ 1 nm similar to ubiquitin)^18^. As noted before, small inorganic nanoparticle systems have likewise been found to exhibit increasingly large values of 〈*u*^*2*^〉 in their cores upon approaching their melting transition *T*_*m*_ from below^33^. Since water apparently destabilizes the folded state of ubiquitin in comparison to glycerol, the increase 〈*u*^*2*^〉 in the core of ubiquitin is just the expected trend from the protein-inorganic NP analogy for small NPs destabilized by an alteration of environmental conditions, such as adding water. We next consider changes in local mobility with the addition of water from the perspectives of the tertiary and primary structures of the protein.

**Figure 4:**
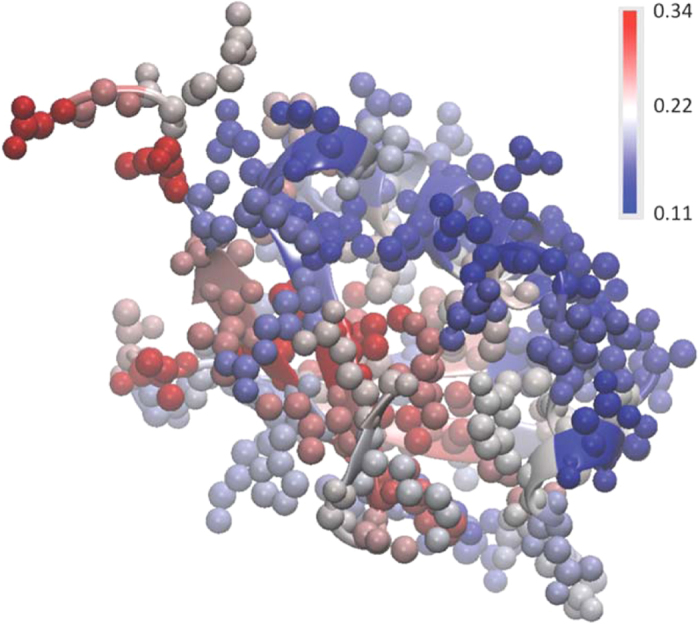
Ubiquitin atoms in the glycerol solution are color-coded by their values of 〈*u*^2^〉 in units of Å^2^. All protein atoms, including the H atoms, are included in this analysis.

Figure 4 depicts the magnitudes of 〈*u*^2^〉 for the ubiquitin atoms in the glycerol solution in real configuration space. The values of 〈*u*^2^〉 are ‘patchy’, i.e., there are relatively stiff regions separated by soft regions. The fraction of stiff regions in the protein (low 〈*u*^2^〉) diminishes with an increase in the amount of water. This effect, which amplifies on the observations of Fig. 2(b), is described in the Supplementary Information. In the language of GF liquids, the protein dynamics is clearly ‘dynamically heterogeneous’.

**Figure 5:**
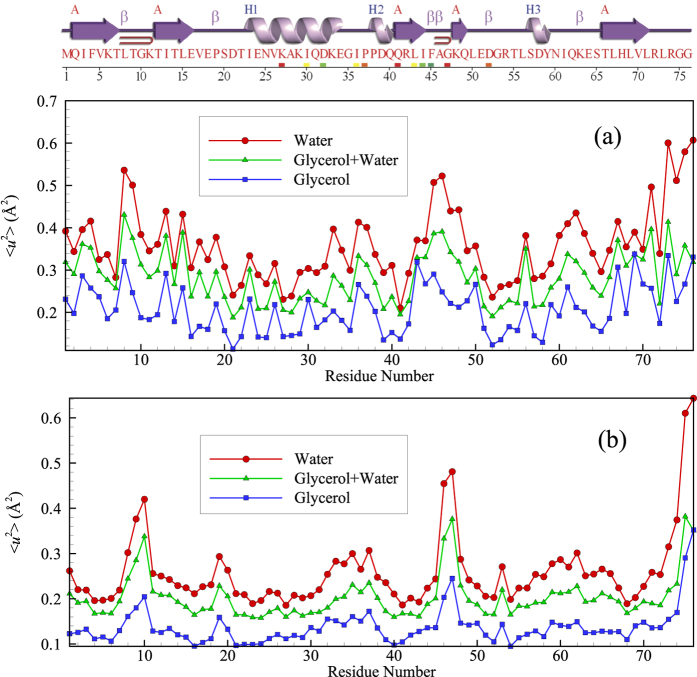
(**a**) Variation of average 〈*u*^2^〉 values of all carbon atoms per residue along the sequence of ubiquitin. (**b**) Variation of 〈*u*^2^〉 along the sequence of the C_α_ atoms of ubiquitin. Top figure exhibits ubiquitin’s secondary structure. The present simulations qualitatively accord with experimental estimates of the C_α_ atoms 〈*u*^2^〉 for ubiquitin as a function of residue number^50^. We show both the alpha carbon and all the carbon atoms of the protein because we find that a description of the collective atomic motion within the protein requires that *all* of the carbon atoms to be considered rather than just those of the protein backbone. Many experimental and computational studies focus on just the alpha carbons.

Some additional insight into variations of local mobility can be obtained by comparing 〈*u*^2^〉 as function of the primary protein sequence, with reference to the location of the ordered alpha helix and beta sheet domains within the protein. Figure 5 represents the average 〈*u*^2^〉 for all carbon atoms in each residue as well the values for only the alpha carbon atoms. We see that 〈*u*^2^〉 tends to be substantially larger in regions between the α helical and β sheet regions of the protein, as might naturally be expected. These ordered regions of the protein are evidently analogous to crystal grains in polycrystalline materials, which are likewise characterized by relatively low 〈*u*^2^〉 values compared to the grain boundary domains surrounding them. Since the grain boundaries in metallic material (Ni) are found to exhibit collective dynamics similar to that of GF liquids^22^, we next describe how the variation of solvent composition affects the collective dynamics within the relatively disordered regions of the protein.

### String-Like Collective Atomic Motion in Ubiquitin

Cooperative particle dynamics presents one of the most characteristic features of the dynamics of GF fluids^28^,^63^,^64^. In addition, there is intense recent interest in the role of conformational fluctuations and cooperative atomic motion in the catalytic behavior of proteins^65^,^66^,^67^,^68^. Following standard procedure in the field of GF liquids, the first step in identifying collective particle rearrangement motion involves identifying the ‘mobile’ atoms in the system^28^ by comparing the self-part of the van Hove correlation function *G*_*s*_(*r*) to that for an ideal uncorrelated liquid exhibiting Brownian motion, a system for which *G*_*s*_(*r*) reduces to a simple Gaussian function. *G*_*s*_(*r*) for an interacting fluid possesses a long tail at large distances *r*, indicating the existence of particles with relatively high mobility in an interacting particle system. A comparison of this kind generally produces a crossing of the *G*_*s*_(*r*) curves for the interacting and non-interacting systems, and the mobile particles are then naturally defined as those atoms whose displacements exceed the distance at the crossing point after a characteristic diffusive decorrelation time, Δ*t*. The van Hove correlation function of all of the protein atoms in Fig. 1(a) does not represent a single-peaked function, but rather has *multiple peaks* centered at successive locations. Therefore, we conclude that the ‘mobile’ atoms are essentially those particles moving a distance *r(t*) that exceeds the typical amplitude of an atomic vibration after Δ*t* but that is smaller than a particular distance. Mathematically, these particles are identified by a threshold condition for the atomic displacement, *a* < |**r**_*i*_(Δ*t*) − **r**_*i*_(0)| < *b*, involving constants *a* and *b* that can be determined from the van Hove correlation function. Then, the identification of correlated atom motion requires a consideration of the *relative displacement* of the particles.

**Figure 6:**
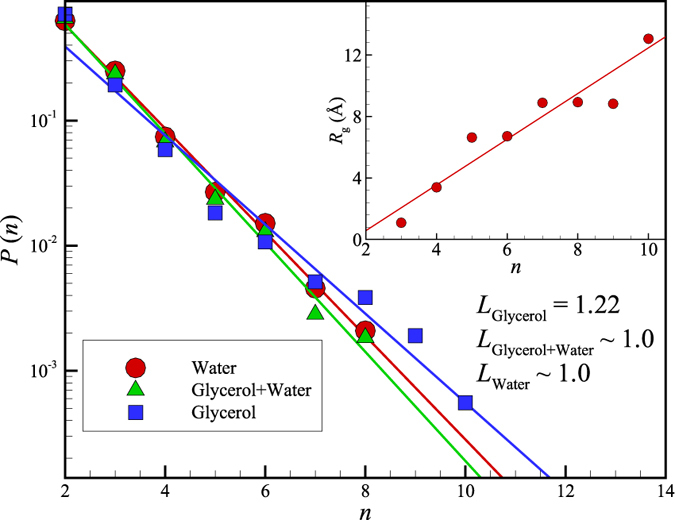
Length distribution of string-like collective rearrangements of the atoms within ubiquitin molecule in glycerol, water, and a glycerol-water mixture at *T* = 300 K. The inset displays the average radius of gyration of the ubiquitin strings, *R*_*g*_, as a function of the average string length *L* = 〈*n*〉 in glycerol, the only case where collective motion is appreciable at this *T* in our CHARMM36 simulations. We see that *L* is near 1 in both the pure water and water/glycerol mixture, indicating that the TIP3P water has essentially suppressed all collective exchange motion within the protein, proving further evidence that the protein dynamics is ‘slaved’ to the dynamics of the solvent. The average radius of gyration *R*_*g*_ describes the average spatial extent of the dynamic clusters, while *L* describes their average contour length. Note that the strings are highly polydisperse, which tends to make the average values of *R*_*g*_ and *L* rather small in comparison to larger strings seen in a visualization of collective atomic exchange events within the protein.

Collective atomic motion implies that the spatial relation between the atoms is preserved to some degree as the atoms move. Specifically, the reference mobile atoms *i* and *j* are considered to be within a collective atom displacement *string* if they remain in each other’s neighborhood, and we specify this proximity relationship by, min[|**r**_*i*_(Δ*t*) −**r**_*j*_(0)|, |**r**_*i*_(0) −**r**_*j*_(Δ*t*)|] < 1.0 Å. Atomistic simulations of GF liquids indicate that the distribution of string lengths *P(n*) is approximately an exponential function of the number of atoms in the string *n*,


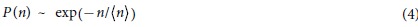


where *P(n*) is the probability of finding a string of length *n* at the characteristic time, t*. We examine the length *n* of the strings over a time interval t* and make a histogram of the string length t* to obtain *P(n*). We repeat this procedure for a number of t* intervals to achieve better statistics. Note that the ‘string length’ *n* is dimensionless as it involves the number of atoms participating in the string. The strings are highly polydisperse in length and we characterize the length by *L*, the mean value of *n* determined from the string length distribution. Eq (4) implies that the average string length 〈*n*〉 = *L* can then be determined from the slope of the fitted lines in Fig. 6. The average string length determined in this way is estimated to equal *L* = 1.22 ± 0.05 for glycerol, but no appreciable collective exchange motion within ubiquitin for TIP3P water at this temperature was observed, i.e., *L* ≈ 1. Evidently, the cooperative motion within protein atoms is suppressed when water is added.

While the characteristic time t*, the average lifetime of the string excitations, ranges between 1 *ps* to 50 *ps* in the simulation data shown in Fig. 1, this time, along with the segmental relaxation time of the protein *τ*_*α*_ obtained from the intermediate scattering function [Fourier transform of van Hove function, *G*_s_(*r, t*); See Supplementary Information], become much larger at lower temperatures. Because of this slowing down of the protein and solvent dynamics upon cooling, it becomes progressively difficult to perform equilibrium simulations at much lower temperatures, as in the case of glass-forming systems generally (See Supplementary Information). Recent modeling of the strings in GF liquids as equilibrium polymeric structures, however, has allowed for an extrapolation of simulation observations of structural relaxation time *τ*_*α*_ determined at higher temperatures to low temperatures where direct molecular dynamics simulations are not currently possible^69^.

**Figure 7:**
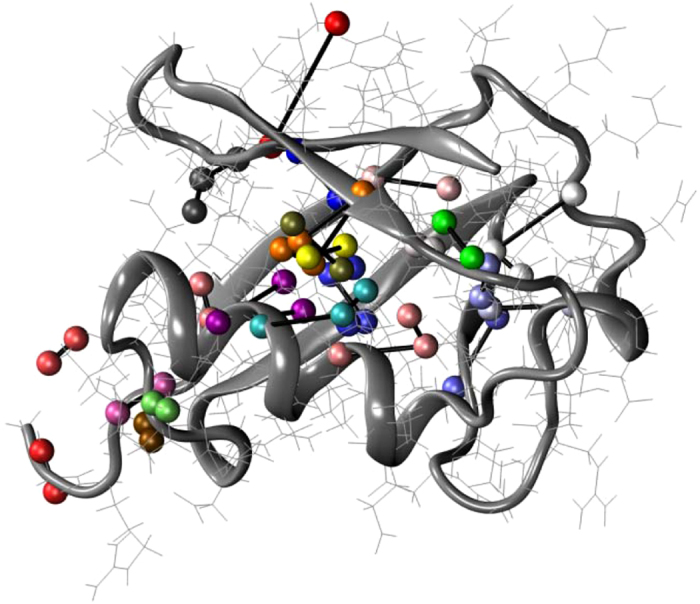
Representative strings of collective motion within ubiquitin in glycerol near room temperature where the protein backbone is displayed as a ribbon diagram. Ubiquitin atoms belonging to a common string are shown as spheres with same colors and are connected by bars. The strings are physically localized in the ‘disordered’ (loop) regions of the protein between the relatively well organized and dynamically inactive alpha helical and beta sheet regions. The concerted exchange motion within the protein is then concentrated between secondary structure elements of the protein. The concentration of collective atomic exchange motion to relatively disordered grain boundary regions between crystal grains is a characteristic of polycrystalline materials^22^. In the discussion section, we discuss recent experimental studies that have indicated that collective motion within proteins is constrained to “channels” defined by the secondary structure of the protein, a physical picture that we think is consistent with our simulation observations.

We now apply the measures of collective motion developed previously in the context of GF liquids (i.e., ‘cooled’ liquids showing collective motion characteristic of incipient glass formation) to the dynamics of ubiquitin molecule in glycerol, water and a glycerol mixture at room temperature, with the expectation that water ‘plasticizes’ the protein dynamics, thereby reducing the scale of collective motion. Just as for the Ni NPs [See Fig. 2 of ref. 4], highly collective molecular motions emerge in the form of string-like atomic rearrangements in our protein systems (Figs 6 and 7). These motions are much more prevalent in glycerol at room temperature (*T* = 300 K) according to the measures of dynamical heterogeneity noted above (e.g., the average string length *L*). While the addition of water increases the average amplitude of local fluctuations in atomic position within ubiquitin at room temperature by lowering the glass transition temperature of the solvent (water plasticizes the glycerol solution^70^), the scale of collective motion *L within* the ubiquitin molecule is correspondingly attenuated. We suspect that this reduction of collective atomic motion within the protein reflects a corresponding reduction of the length of solvent molecule strings *L*(solvent) that drive ubiquitin atomic motion, but this hypothesis needs to be checked by explicit computation of both *L*(solvent) and the spatial correlations between the ubiquitin and solvent strings. Little is known currently about the molecular nature of coupling between the collective dynamics of macromolecules and the solvent. Molinero and Goddard^71^ have made an interesting simulation study of the concerted atomic motion of water and much larger glucose molecules (Note the similarity of the water van Hove function *G*_s_(*r, t*) in Molinero and Goddard with our observations for ubiquitin in Fig. 1). Neutron scattering studies have inferred the existence of clusters of correlated atomic motion in proteins on a *ps* timescale^29^. In particular, Bu and coworkers^29^ found that the spatial scale of the correlated motions diminished in the folded state compared to the protein in its unfolded state, a state reached by raising the temperature or the addition of a ‘denaturant’ that shifts the folding temperature downward. Specifically, they inferred the presence of string-like collective rearrangement motion with the spatial extent of the strings equal to 1.8 (±0.4) *nm* and 6.9 (±0.12) *nm* in the native and unfolded states of bovine α-lactoglobulin at 30 °C, respectively. While these scales are qualitatively in line with our estimates of string *R*_*g,*_ in Fig. 6 for collective motion in the native and unfolded states of ubiquitin, respectively, the methodology for estimating the string length from the neutron data requires a close examination and further validation. Nonetheless, we are encouraged by these preliminary results.

As noted before, ubiquitin resembles a polycrystalline NP with ‘grains’ corresponding to alpha helices and beta sheets organized within these naturally occurring nanoparticles. As with polycrystalline metallic materials such as Ni bi-crystals^22^, we expect collective motion to be localized at the boundary regions separating the alpha helices and beta sheets rather than exclusively being on the NP surface, an expectation amply confirmed by Fig. 7. Simulations of ice nanoparticles produce a similar polycrystalline internal grain structure, presumably due to a common frustration in molecular packing induced by competing hydrogen bond interactions^72^. This type of inorganic NP evidently serves as a better analog of folded proteins than our Ni NPs.

**Figure 8:**
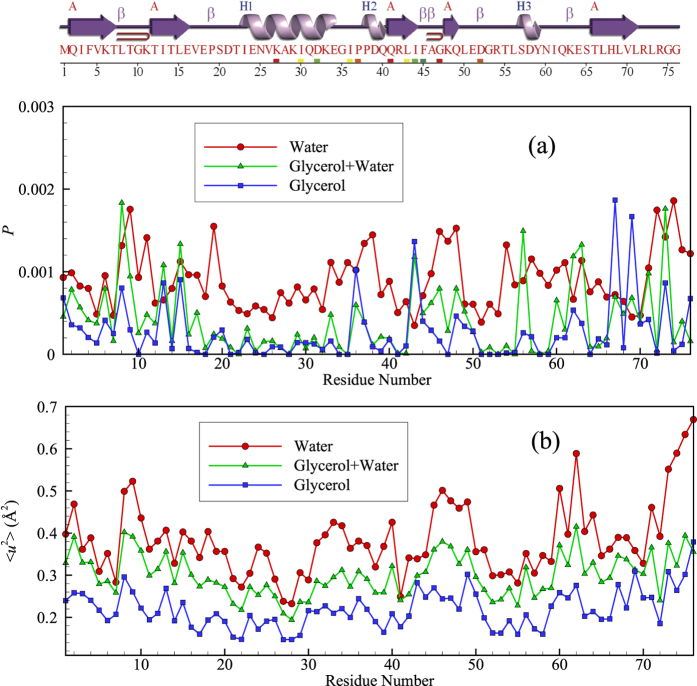
Comparison of the magnitude of 〈*u*^2^〉 of the all atoms (excluding hydrogen atoms) averaged per residue and the probability *P* that the residue atoms are participating in a string motion at any time (averaged over the residue atoms). The amino acid type along the sequence and the position of the secondary structural elements are indicated at the top of the figure.

Although it is clear from Fig. 7 that the string-like collective motion is largely confined to the disordered protein regions (loops) separating the alpha helices and beta sheet domains, we can further quantify this effect by examining the location of the string-like collective motion within the protein from the standpoint of the secondary structure of the protein. Figure 8 considers a direct comparison of the probabilities that atoms of the protein (excluding hydrogen) are involved in a string-like displacement over the course of simulation to the magnitude of 〈*u*^2^〉 for these atoms along the amino acid sequence for glycerol, glycerol-water and water solutions. Ubiquitin’s secondary structure is indicated at the top of the figure to help visualize the locations of the strings. It is clear that regions of relatively large 〈*u*^2^〉 are also regions in which string-like collective motion tends to be localized. This is exactly the effect observed previously for the grain boundaries of Ni and in Ni NPs^13^,^22^, and in the interfacial dynamics of bulk crystalline Ni^14^. This finding is natural since the strings are the manifestation of highly anharmonic intermolecular interactions that must be prevalent in the protein domains between the α-helices and β-sheets.

### Quake-like Protein Motions and Colored Noise

Previous studies of the dynamics of proteins indicate the occurrence of quake-like changes in molecular configuration^73^, and recent reports of single-molecule measurements describe intermittency in the internal motion of proteins that can follow fluctuations in the positions of residues within individual proteins^74^,^75^. It is natural to expect this type of phenomena to be related somehow to dynamic heterogeneity and the string-like collective motion indicated above, so we next examine this aspect of the dynamics of ubiquitin.

MD simulations of Ni NPs under surface-melting conditions indicate that the string-like collective motion is accompanied by colored noise in the frequency spectrum of the fluctuations in particle displacement, i.e., 〈*u*^2^〉. (A similar power law scaling of the intensity of the fluctuations is also observed in earthquakes)^13^. Given the many similarities of the dynamics of inorganic nanoparticles to proteins, we examine if these phenomena also arise in the dynamics of ubiquitin.

**Figure 9:**
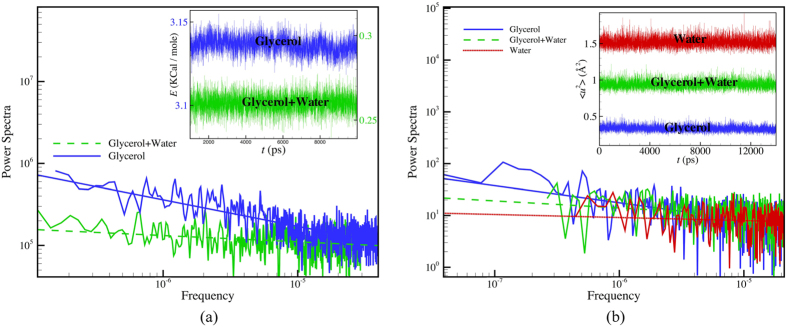
(**a**) Power spectrum of the potential energy fluctuations for ubiquitin and solvent at *T* = 300 K in glycerol and in glycerol/water solutions. The glycerol data exhibit a noticeable power-law scaling with frequency, *P*( *f*  ) ∼ 1/*f*^***α***^ where α = 0.32 ± 0.02, but the protein potential energy fluctuations in the pure aqueous solution are nearly white, i.e., α ≈ 0. (**b**) Power spectrum of 〈*u*^2^〉 fluctuations of the all atoms of ubiquitin in glycerol, a glycerol/water mixture and water at *T* = 300 K. The inset shows a representative portion of the energy and 〈*u*^2^〉 times series from which the power spectra are derived (all the simulation data was used to calculate the power spectra; energy values are NAMD output energies).

Our examination of fluctuations in ubiquitin in glycerol, glycerol/water and water (Fig. 9) first focuses on the fluctuations of the entire system potential energy, composed of *both* the solvent and protein. The Fourier transform of the time series displayed in the inset to Fig. 9(a) indicates that the power spectrum of the noise for the system potential energy fluctuations in glycerol solution takes a power-law form, i.e., *P(f*) ∼ 1/*f*  ^α^, to a good approximation with a power of 0.32 ± 0.02, while the power spectrum becomes nearly flat, i.e., α = 0, after water is added to the glycerol solution (The power spectrum *P(f*) of these fluctuations is defined by the transformation, *P(f*) = |∫ *E(t)e*^−2π*ift*^ *dt*|^2^ where *E(t*) is the time series of the energy fluctuations. A power spectrum of the noise exhibiting a power law scaling, *P(f*) ∼ 1/*f*  ^α^, is said to be ‘colored’. ‘White noise’ corresponds to α = 0, ‘red’ noise to α = 1 and intermediate α defines “pink” noise). This change in dynamics parallels the reduction in the scale of collective motion with the addition of water to the glycerol solution described above. Many previous works have emphasized the occurrence of power law fluctuations in the potential energy of folded proteins^76^,^77^,^78^ and in water, methanol and silica^79^,^80^ so there is no need to dwell on this phenomenon further here^76^,^77^,^78^. Associated long-range temporal fluctuations in fluorescence intensity, reactivity, etc., of both nanoparticles and proteins have also been observed experimentally^81^,^82^,^83^,^84^,^85^,^86^.

Since the energy fluctuations in the solvent are large, one might expect the power-law scaling of the potential energy fluctuations of ubiquitin to arise mainly from the solvent driving the protein motion. We then focus only on fluctuations within the ubiquitin molecule alone. In particular, we consider fluctuations in mobility and thus fluctuations in 〈*u*^2^〉 (this quantity is determined at each *ps* of simulation time). This ‘fast dynamics’ averaging time is comparable to the measurement time of 〈*u*^2^〉 in a neutron scattering measurement and physically corresponds to a molecular caging time^30^. Figure 9(b) shows 〈*u*^2^〉 fluctuations for the protein atoms, i.e., all atoms considered without discrimination. In glycerol solution, we again observe evidence for highly colored noise in the power spectrum of the 〈*u*^*2*^〉 time series, but there seem to be systematic oscillations in the data that unfortunately do not allow a reliable estimate for the noise exponent α. However, we see that the introduction of water leads to a nearly ‘white’ spectrum (*α* = 0) for the 〈*u*^2^〉 time series so that we again have evidence that the extent of correlations in the fluctuations becomes greatly diminished upon adding water. Our previous analysis of the 〈*u*^2^〉 fluctuations in the interfacial dynamics of Ni nanoparticles indicated a direct relation between the α and the average string length, *L*, i.e., *α* = *L* − 1^30^. In the present study, we find that a similar relation between *α* for the potential energy fluctuations and *L* holds qualitatively for ubiquitin, i.e., *L* − 1 = 0.22 ± 0.05 versus *α* = 0.32 ± 0.02 for glycerol solution, while, *L* − 1 ≈ *α* ≈ 0, for the aqueous solutions. Our previous studies of the noise exponents *α* describing potential energy and 〈*u*^2^〉 fluctuations indicate that these exponents are not always equal^14^,^30^ and the exact relationship between *L* and *α* in ubiquitin solutions requires a systematic variable temperature and composition study.

**Figure 10:**
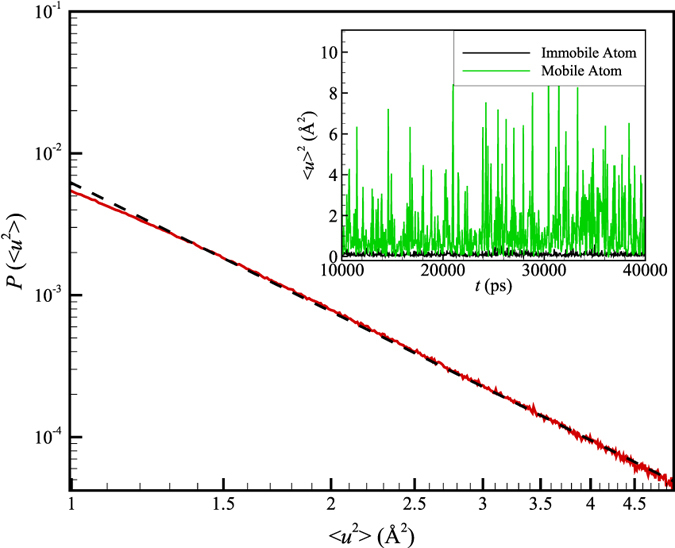
Probability distribution function *P*(〈*u*^2^〉) for the magnitude of peak values of 〈*u*^2^〉 for mobile ubiquitin atoms (atoms with 〈*u*^2^〉 values larger than 1) involved in collective string-like motion in the glycerol solution. Dashed line is the fitted power law and the red solid line indicates simulation observation. Previous observations^13^ of quake-like displacements in the interfacial dynamics of Ni NPs are similar [See Fig. 4 of ref. 13]. Inset shows the quake-like displacements from which the distribution *P*(〈*u*^2^〉) was obtained.

The inset of Fig. 10 shows a time series of the intensity fluctuations in 〈*u*^2^〉, i.e., peak values of 〈*u*^*2*^〉, for the ubiquitin atoms undergoing string-like collective motion in the glycerol solution in comparison to the majority immobile atoms that are not in this ‘active’ dynamical state. We see that the mobile atoms move cooperatively exhibiting large displacement or ‘jumps’ after short time intervals, while the remaining ‘immobile’ atoms undergo relatively small displacements having a small variance. These observations are strikingly similar to our previous observations on the string atoms in the interfacial dynamics of Ni NPs^13^ and the interfacial dynamics of bulk crystalline Ni^14^. As discussed at length in these former works^13^, these fluctuations in displacement also greatly resemble displacement observations made in association with earthquakes where the collective displacements are likewise found to be concentrated in the boundary regions between the earth’s tectonic plates (another type of ‘grain boundary’ region, albeit having a rather different scale than found in proteins and Ni NPs). The power law scaling quake exponent, *γ*, describing the probability distribution of the ubiquitin atom jump size (〈*u*^2^〉) takes a value near 3.0 ± 0.05 at room temperature when glycerol is the solvent (Fig. 10), a value similar to that found in our former Ni NP observations^13^ where *γ* was found to be somewhat dependent on temperature. In this respect, the dynamics of ubiquitin is remarkably similar to the dynamics of Ni NPs. In our previous study of *γ* in the context of the interfacial dynamics of crystalline Ni, the exponent *γ* was found to be inversely related to the colored noise exponent, i.e., *γ = *1/*α,* to a high approximation, where *α* describes *both* the potential energy and 〈*u*^2^〉 fluctuations of the interfacial atoms exhibiting glassy dynamics. Since the potential energy noise exponent *α* for ubiquitin in glycerol was estimated above to equal *α = *0.32 ± 0.02 (system potential energy), an inverse relation between *α* and *γ* found in previous work is supported by our ubiquitin simulations to within numerical uncertainty.

## Conclusions

The idea that a protein in its biologically active native state behaves as an amorphous solid, i.e., ‘glass’, can be traced back to Schrödinger^1^,^87^. Apart from observations consistent with this hypothesis indicating that the protein density^88^ is overall similar to that of solid hydrocarbons^89^,^90^, this hypothesis has been interpreted as having implications regarding the *dynamics* of proteins^1^. We indeed observe collective atomic motion in proteins (‘organic nanoparticles’) that is similar in many respects to the pattern of molecular dynamics found in GF liquids. In particular, this correspondence is evidenced by a range of dynamic behaviors, symptomatic of GF liquids- the presence of a secondary peak in the van Hove correlation function *G*_*s*_(*r, t*) for atomic displacements, a growing non-Gaussian parameter upon cooling, string-like collective motion, etc. The fluctuations in the potential energy and in atomic displacement have a colored noise spectrum, implying an even closer analog view of a protein as being similar to an aperiodic solid (‘glass’) particle with a semi-liquid interfacial region^1^,^91^. In accord with the dynamics of inorganic nanoparticles, proteins have a relatively mobile interfacial layer that has important implications for protein function, as discussed by Karplus and coworkers^1^.

The important, perhaps obvious, feature in the dynamics of ubiquitin that makes it different from the idealized “aperiodic solid” or “glass” protein model of Schrodinger and the surface melted nanoparticle model of Karplus and coworkers^1^ is the presence of organized α-helices and β-sheet structures within the protein. These structures are analogous to crystal grains in inorganic nanoparticles. Similar to previous studies of atomic motions in the grain boundary regions of polycrystalline Ni, collective string-like motion is indeed observed for the atoms in the grain boundary-like regions separating α helix and β sheet regions of the protein. We may thus conclude that the dynamics of folded proteins can be considered as being analogous to that of small polycrystalline inorganic nanoparticles, such as those found in recent simulations of ice nanoparticles (objects of great interest in connection with studies of atmospheric chemistry)^72^.

An essential problem in understanding how proteins, and indeed NPs in general, interact with the host medium relates to how the solvent dynamics *couples* to that of the protein. This coupling phenomenon is widely recognized as being crucial for understanding biological function, but limited methods are available for studying the physical nature of this phenomenon at a molecular level. Therefore, we have initiated an investigation of protein-solvent coupling using concepts and metrologies drawn from the field of glass-forming liquids. Our analysis indicates that the addition of water greatly diminishes the scale of string-like collective motion within the protein, and, correspondingly, the noise color for the potential energy fluctuations of the system as a whole decreases. (The noise color is conventionally quantified by the spectral exponent where ‘white noise’ corresponds to a vanishing spectral exponent (*α* = 0), ‘red’ noise corresponds to a 1/*f*  ^*α*^ power spectrum (*α* = 1), and intermediate exponents are said to correspond to “pink” noise). The simulations offer strong evidence indicating the presence of coupling between the dynamics of the protein and the solvent. The methodologies described in the present paper should then provide powerful tools for further exploring this type of coupling.

An important question to pose at this stage is why anyone should be concerned with the existence of collective motion in the form of strings within proteins. Recent studies of a wide range of liquids with colored fluctuation spectra, ranging from polymeric to metallic glass-forming materials^24^,^34^, demonstrate that changes in the activation free energy can be *quantitatively* explained in terms of changes of the average string length, *L*. Moreover, changes in the dynamics of thin polymer films and nanocomposites can be equally well described within a unified framework^56^. Similarly, relaxation processes in proteins characteristically exhibit a non-Arrhenius temperature dependence^29^,^92^,^93^,^94^,^95^, and we may then anticipate that the strings will provide quantification of the ‘dynamic heterogeneity’ and fast collective motion exhibited by proteins. The observation of intermittent quake-like motion^73^ associated with the sub-dynamics of the string motion found in our ubiquitin simulations provides insight into the highly intermittent dynamical^96^,^97^ activity of proteins (“blinking”) revealed by single molecule measurements^65^,^81^. In particular, our observation that the intermittent fluctuations in the potential energy and particle displacements can be tuned by varying the solvent composition also provides a quantification of how the solvent motions couple (‘enslave’) to those of the protein^98^,^99^. We also observe precisely how and where the solvent modifies the collective motion of the protein on a time scale t* related to internal chain segment diffusion within the protein; t* ranges from a timescale on the order of *ps* in our relatively high temperature simulations to a timescale as long or longer than the protein folding time at low temperatures so that t* has a large dynamical range.

### Relevant Observations of Collective Motion in Glass-Forming Materials and Proteins

Recent measurements on the dynamics of proteins at high concentrations show a dramatic slowing down of the relaxation dynamics that is accompanied by a large increase in the solution viscosity that parallels glass-formation in colloidal suspensions^100^. Direct observation of colloidal particle motions in GF liquids^101^,^102^ and in the grain boundaries of polycrystalline colloidal materials^103^ allows for the direct imaging of exactly the same type of collective string motion that we see in the present paper for atomic motions within ubiquitin. Proteins function biologically in a “crowded” environment^104^,^105^ and we may expect a coupling of the collective dynamics *within* the protein to other proteins through collective motion of the protein as a whole on longer time scales. This effect is modulated by the collective motions of the solvent that also couples to the internal and large-scale center of mass motion of the proteins. In the future, it would be interesting to explore the nature of this coupling process^71^ under the congested conditions of the cell interior or the extracellular biological environment.

We can generally expect from previous studies on GF liquids that crowding interactions between proteins, and between proteins and other macromolecules and structures in their environment, to counterbalance the plasticizing effect of water on the protein dynamics^106^,^107^ (exhibited in our simulations) by inducing an enhanced collective dynamics both within the protein and between proteins. Indeed, there is direct evidence that increasing the protein concentration has the effect of suppressing the mean square amplitude of atomic fluctuations averaged over the entire molecule, 〈*u*^2^〉^108^. Recent simulations on glass-forming materials have shown that the scale of string-like collective motion, *L,* scales with 〈*u*^2^〉 to a negative power under a wide range of thermodynamic conditions^109^. Based on the described analogy of proteins and GF material, a decreased amplitude of atomic motion within the protein in more concentrated (‘crowded’) media, such as the cell interior, can naturally be expected to lead to enhanced collective motion (*L) within* the protein. Modulations of protein collective motion in the course of their natural biological function can also be anticipated in association with the binding of proteins to each other, as in the formation of structural elements of the cytoplasm (actin, tubulin intermediate filaments), in amyloid fiber formation or through the process of protein binding to DNA, drugs or other diverse species. This modulation of cooperative motion through binding should be rather universal since molecular binding generally alters molecular rigidity (〈*u*^2^〉) and correspondingly must alter the extent of collective motion within the proteins forming such complexes. New modes of collective motion can also be expected to arise through molecular binding in which the collective motion within one protein extends into the protein or other molecule to which it binds. The investigation of emergent collective effects arising from intermolecular interaction is an attractive topic for future simulation studies.

Large scale string-like collective motion has been observed in simulations of physical aging of polymeric GF materials^110^, the melting and freezing of Ni NP^111^ and the melting of bulk crystalline Ni^23^; the same methodology can also be applied to diverse far from equilibrium dynamic processes of proteins. Further experimental and computational studies are needed aimed at understanding the significance of collective motion for biological catalysis^112^,^113^,^114^ and other essential protein functions^93^.

We finally note that there are some exciting recent measurements on the ‘fast’ dynamics of proteins having sufficiently high spatial and time resolution to allow for qualitative comparison with our work, although these measurements normally correspond to non-equilibrium conditions in which the collective motion is more observationally conspicuous. In particular, the photoexcitation of the binding complex of CO and the heme group of myoglobin has been observed with a spatial resolution on the order of 1 nm and a temporal resolution of better than 100 *ps*^115^,^116^,^117^,^118^,^119^,^120^. The resulting ‘movies’^117^,^121^ showing the protein response to this type of optical excitation have revealed that the atomic motions involve highly correlated motions with a damped inertial character. These collective atomic motions involve amino acid residues located in the ‘liquid-like’ loop regions between the rigid structural elements of the protein (i.e., alpha helices and beta sheets). In particular, following the initial impulsive event of photoexcitation^121^,^122^,^123^, the resulting stress is relaxed through a propagating molecular disturbance within the loop regions on the length scale of the individual residues where the process is reminiscent of “falling dominos”^115^. This results in the concerted movement of many amino acid residues “providing a conduit for the transduction of reaction forces to longer length scales to drive functionally relevant protein motions”^116^. Recent studies having a similar high spatial and temporal resolution have shown similar propagating collective motion processes in other proteins such as albumin, photoactive yellow protein and cytochrome c^122^,^124^,^125^. Miller *et al*. go on to suggest that these ‘fast’ correlated motions define the reaction coordinates that predominantly govern the dynamic exploration of the protein configuration space, preventing the protein from getting stuck for too long in deep potential minima that exist by virtue of strong attractive directional intermolecular interactions within the class of globular proteins. They have similarly characterized these photo-induced motions as being similar to ‘earthquakes’ because of the intermittency of motion and the collective sliding events involved. These observations broadly accord with our observations of collective exchange motion within ubiquitin where the coordinated motions are confined to regions of the protein that not in the relatively rigid alpha and beta sheet regions, the ‘loop regions’. Miller and coworkers^121^ suggest this type of collective, almost ballistic, motion on short timescales is highly functional in essential protein activities such as catalysis and intermolecular signaling and that protein structure is highly evolved to precisely direct these energy transduction channels for these purposes. Evidently, mutations or molecular binding in the loop regions should modulate these coordinated motions, leading to allosteric alterations in protein function. Williams and Dermott^126^ have provided experimental evidence that conformations motions in these loop regions indeed have direct significance in enzyme catalysis. We further note that recent computational studies of changes of stability and long range changes of mutant antibodies seem to involve amino acid sequence changes that occur almost exclusively in the loop regions^127^ (See Fig. 1 of the paper).

Kong and Karplus^128^,^129^ have also uncovered evidence from molecular dynamics simulations for this type of cooperative motion and allosteric modulation. In particular, they studied PDZ domains of a model signaling protein (Protein databank ID 3 PDZ) and found collective motion to be localized in “loop” or “hinge” regions between the alpha helices and beta sheets (Compare Fig. 1 of Kong and Karplus and Fig. 8 of the present work where we see that the protein regions exhibiting “strong displacement correlations” and large Debye-Waller factor are localized in similar protein regions in ubiquitin as in this PDZ protein). The PDZ domains are believed to be essential “signal transduction pathways” that are “imprinted on the (protein) structure by evolution” to affect allosteric signaling within and between proteins, acting as an essential link in the network of collective interactions that link motions at an atomic scale to macroscopic organism response^130^,^131^,^132^.

We suggest that the strings that we investigate exactly correspond to the fast collective motions observed in recent simulations and measurements of proteins under equilibrium conditions, as in the simulations of Kong and Karplus, and under non-equilibrium conditions of excitation by external stimulus, such as photoexcitation or mechanical stress, so that our analysis of collective motion should provide a useful tool in quantifying these collective motions in connection with diverse critical protein function in living systems.

### Simulation Details

Three different molecular dynamics simulations have been run using NAMD 2.9^133^ and the CHARMM36 force field on the Midway and Beagle clusters at the University of Chicago. The simulated systems are ubiquitin (PDB id 1UBI; 76 residues) in pure TIP3P water model, ubiquitin in pure glycerol, and ubiquitin in an 85% glycerol by volume glycerol-TIP3P water mixture. The force-field parameters for glycerol are derived using the lipid parameters of Charmm36 and are very similar to the values obtained by Reiling *et al*.^134^ using Charmm22. Initially a box of 2000 glycerol molecules (62 × 62 × 62) *Å*^3^ is created and energy minimized to remove strict clashes, and then the system is gradually heated to 300 °K in 0.55 *ns.* The glycerol box is equilibrated at this temperature and constant pressure (1 bar, isobaric−isothermal ensemble) for 20.25 *ns*. The average calculated density inside the glycerol box during the last 8 ns of the equilibration of 1.16123** **g/m^3^ (with a standard deviation of 0.006 g/m^3^) is smaller by 7.7% than experimental [At 25 **°**C, the density^135^ of glycerol is 1.25802 g/cm^3^]. The temperature fluctuates by about ±4° during the glycerol box equilibration. Variations of ≈4.5% to ≈9% have been reported for glycerol density using other force fields^43^. A similar protocol is used to prepare the 85% glycerol by volume glycerol-water mixture system, which is equilibrated for 19.8 *ns*.

Ubiquitin was added to the pure glycerol and glycerol-water boxes after the equilibration. All systems are first energy minimized to remove strict clashes and then are gradually heated to 300 K while keeping the protein backbone restrained. The constraints are gradually removed during the equilibration period of 2 *ns*, and trajectories are run for each of the three systems for totals of 56.4 *ns* (IUBI water system), 60 ns (IUBI glycerol-water system), and 88.9 ns (IUBI glycerol system). No large-scale conformational changes of ubiquitin appear during the simulations.

To compare the behavior of ubiquitin to other proteins, we simulated two other proteins, insulin degrading enzyme (IDE, PDB id 4IOF, chain A; 990 residues) that is a very large protein with a cavity at its core and the bovine serum albumin (PDB id 4FSS; chain A; 583 residues). The simulation was performed using explicit TIP3P water model, and the simulation detail was similar to the ubiquitin as described above. The IDE was run for 1 *μs* and the bovine serum albumin for 63 *ns.*

Our constant pressure (1 bar) and constant temperature *T* = 300 K (NPT ensemble) simulations were regulated using Nose**-**Hoover Langevin piston pressure control^136^,^137^ and Langevin damping dynamics^138^. Periodic boundary conditions and water wrapping were applied. Bonded and short-range non-bonded interactions are calculated at every time step (1 *fs*) and every other time step, respectively. Electrostatic interactions are evaluated at every fourth time step using the particle mesh Ewald method^139^. The cut-off distance for non-bonded interactions is 1.2 *nm* (employing a smoothing function). The pair list for non-bonded interaction is calculated every 20 time steps for those pairs with pair-list distances ≤13.5 *Å*.

## Additional Information

**How to cite this article**: Haddadian, E. J. *et al*. Comparative Study of the Collective Dynamics of Proteins and Inorganic Nanoparticles. *Sci. Rep.* **7**, 41671; doi: 10.1038/srep41671 (2017).

**Publisher's note:** Springer Nature remains neutral with regard to jurisdictional claims in published maps and institutional affiliations.

## Supplementary Material

Supplementary Information
