# Supplementary material for: Comparative Study of the Collective Dynamics of Proteins and Inorganic Nanoparticles
Source: Sci Rep. 2017 Feb 8;7:41671. doi: 10.1038/srep41671 (PMC5296861; doi:10.1038/srep41671)
Supplement: Supplementary Information [file srep41671-s1.pdf]

# Supplementary Information: Comparative Study of the Collective Dynamics of Proteins and Inorganic Nanoparticles

*Esmael J. Haddadian[1], Hao Zhang[2], Karl F. Freed[3], Jack F. Douglas[4]*

[1]Biological Sciences Collegiate Division, University of Chicago, Chicago, IL 60637, USA

[2]Department of Chemical and Materials Engr., University of Alberta, Alberta, T6G 1H9 Canada

[3]Department of Chemistry and James Franck Institute, University of Chicago, Chicago, IL 60637, USA

[4]Materials Science and Engineering Division, Materials Measurement Laboratory, National Institute of Standards and Technology, Gaithersburg, Maryland 20899, USA

## A. Dynamics of Water, Glycerol and Water-Glycerol Mixtures Based on CHARMM36 Force Field

Usually intermolecular potentials are ranked based on their capacity to recover basic equilibrium properties such as density, compressibility, etc. or simple dynamic properties, such as the diffusion coefficient ($D$) or shear viscosity $\eta$ over limited temperature range. [1] In a study relating to the cooperative dynamics of proteins and to the coupling of the dynamics of the solvent and the protein, however, we must be particularly concerned about how well our intermolecular potential describes the dynamics of the solvent. Since the water potential is central to CHARMM force field, and any other family of potentials aimed at modeling the dynamics of biological macromolecules, we consider in this section some essential dynamical properties of the modified TIP3P water model that are relevant to assessing dynamical properties of proteins and other biological macromolecules using CHARMM36.

It is well known that $D$ and the shear viscosity[2, 3] of TIP3P deviate from real water at room temperature by a factor ≈ 3, but the large deviation in the calculated melting temperature of water[1] signals a rather serious problem for simulating dynamics, as discussed in the main text.

We now directly confirm this expectation by quantifying the dynamic heterogeneity in the CHARMM36 TIP3P water model near room temperature ($T$ = 300 K) and at a $T$ much lower than the freezing temperature ($T_m$) of real water ($T$ = 250 K). The non-Gaussian parameter $\alpha_2(t)$, defined in the main text in relation to the dynamics of ubiquitin, provides a simple way to calculate this quantity. As a direct observable measure of dynamic heterogeneity [4] $\alpha_2(t)$ can be used to assess the suitability of this potential to describe the dynamics of water and aqueous protein solutions. In Figure S.1(a), we show $\alpha_2(t)$ as function of $t$ for the modified TIP3P model over the range of temperatures indicated in the figure. No detectable peak in $\alpha_2(t)$ is found at any finite time so that this water model exhibits no appreciable heterogeneous dynamics over this temperature range. These results for TIP3P water are contrasted with those for SPC/E water, which are shown for comparison in the inset to Fig. S.1. [5] In contrast to TIP3P, SPC/E water exhibits a dynamic heterogeneity peak in $\alpha_2(t)$ near room temperature and we see that the height of this peak grows sharply upon cooling, a characteristic behavior of glass-forming liquids. [6] The absence of dynamic heterogeneity and collective motion found in our simulations of ubiquitin in TIP3P water directly reflects the absence of dynamic heterogeneity in this water model that would result to the lack dynamical coupling of the of the protein to this solvent. We then suggest that TIP3P should be considered a hypothetical solvent in which collective motion is completely absent rather than as a model of real water.

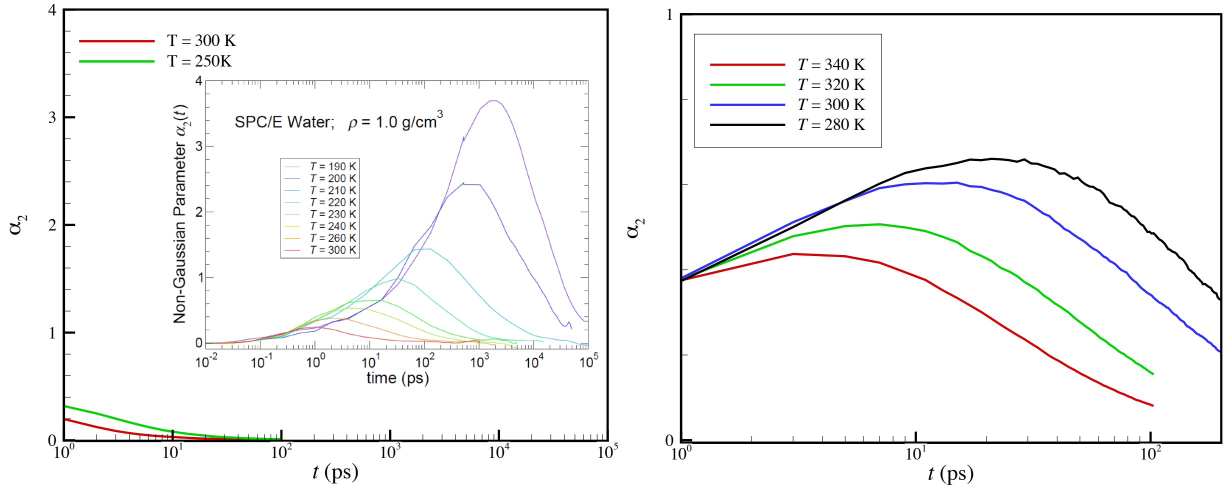


**Figure S.1.** (a) Non-Gaussian parameter $\alpha_2$ of CHARM 36 / TIP3P water near room temperature ($T$ = 300 K) and a representative much lower temperature ($T$ = 250 K) lower than the $T_m$ of real water. We see no detectable peak in $\alpha_2(t)$ at a finite time in this TIP3P water model so that this fluid model is essentially *devoid of dynamic heterogeneity* over this $T$ range. The inset shows corresponding $\alpha_2(t)$ estimates for SPC/E water derived from a previously published study of this water model by Starr and coworkers.[5] Note that $\alpha_2(t)$ for SPC/E exhibits a peak at all $T$ below room temperature. The peak height $\alpha_2(t)$ for SPC/E water is not large near room temperature, making this model more acceptable at this $T$. (b) Non-Gaussian parameter $\alpha_2(t)$ for the CHARMM36 glycerol potential, where a peak was observed at all $T$, suggesting dynamic heterogeneity over this $T$ range.

Fortunately, the CHARMM36 potential for glycerol provides a better description of the dynamics of real glycerol than it does for water. In Figure S.1(b), we show $\alpha_2(t)$ as function of $t$ for the glycerol model over the range of temperatures. Glycerol exhibits a dynamic heterogeneity peak in $\alpha_2(t)$ in all temperatures and the height of this peak grows upon cooling. It is also convenient for our discussion that the glassy dynamics and thermodynamics of CHARMM36 glycerol have recently been reviewed by Jahn et al. [7] As found for glass-forming liquids generally, the $T$ dependence of $D$ follows a *universal* $T$ dependence that is described by the Vogel-Fulcher-Tammann relation (VFT),[8, 9] $D = D_0 \exp [- D_D T_0 / k_B (T - T_0)]$, where $D_0$ and $D_D$

are materials-specific constants, and $T_0$ is a widely reported characteristic temperature of glass-forming liquids. In particular, Jahn et al. [7] find a rather good agreement between CHARMM estimates of $D$ for glycerol over a large $T$ range: they estimate $T_0$ = 180 K for that is reasonably close to the experimental estimate, $T_0$ (expt.) = 170 K. [10] We note for reference that the glass transition temperature $T_g$ and the melting temperature $T_m$ of glycerol are estimated to $T_g$(expt.) = 190 K and $T_m$ (expt.) = 290 K , respectively. Room temperature is then close to melting temperature of glycerol so that significant dynamic heterogeneity can be expected in both simulated and real glycerol near this temperature [See Chen et al. [11] and refs. 7, 12 for a discussion of the characteristic glass transition temperatures for glycerol/water mixture and for an extensive characterization of the dynamics of glycerol-water].

The growing of the structural relaxation time $\tau_\alpha$ with cooling is perhaps the most basic feature of a glass-forming liquid and we briefly consider the CHARMM36 predictions for $T$ dependence of $\tau_\alpha$. Following standard practice, we obtain $\tau_\alpha$ from the decay of the self-intermediate scattering function $F_s(q,t)$, which is just the Fourier transform of $G_s(r, \Delta t)$. Fig. S.2 shows $\tau_\alpha$ estimates deduced from the decay of $F_s(q,t)$ for water and glycerol at the designated $T$. Note that the sharp growth of $\tau_\alpha$ upon cooling. This rapid growth of $\tau_\alpha$ makes equilibrium simulations difficult since longer computational times than $\tau_\alpha$ are required to achieve equilibrium. This basic feature of glass-forming liquids inherently limits the $T$ range that can be simulated by molecular dynamics simulation methods.

The dynamic heterogeneity that we observe for ubiquitin in CHARMM36 glycerol resembles the typical dynamics of a glass-forming liquid, but this type of heterogeneous dynamics is not observed for ubiquitin dissolved in TIP3P water. This significant change in the dynamics of

ubiquitin derives from a strong *coupling* between the dynamics of protein and the solvent. Isolated macromolecules, even idealized ones having homogeneous chemistry and no solvent, can exhibit heterogeneous dynamics and glass-formation[13] so this solvent effect is striking. Just how does the solvent regulate the dynamical heterogeneity and collective dynamics of the protein and why does the "glass transition" of most proteins occur generally in a narrow temperature between 200 K to 220 K, a temperature near the characteristic temperature $T_c$ of pure water, is not known.[14, 15] Although this remains a fundamental question, we can make some informed speculation about this basic phenomenon that pertains to why it is so crucial to have a water model with realistic heterogeneous dynamics.

The hydrating layer of water about the proteins 'dresses' them in a fashion similar to solvation shells about ions in solution. This 'bound' layer of water can be expected to create a cloaking effect[16-18] in which mutual interactions between the amino acid groups of the protein are significantly influenced by this water layer. The cohesive interaction strength is the primary parameter governing the characteristic temperatures of glass-forming liquids[19] and a rather uniform cohesive interaction would make proteins having a similar inherent flexibility and size to have a similar glass transition. Protein hydration could then naturally explain the underlying thermodynamic rationale for why the protein dynamics becomes coupled ("slaved") to the dynamics of the solvent, regardless of the degree of cooperative motion exhibited by the solvent. This situation seems to be evidenced in our simulations of ubiquitin in TIP3P water where we find that ubiquitin exhibits no discernable collective exchange motion, a behavior directly reflected in the nearly perfectly uncooperative dynamics of TIP3P water. Of course, there is substantial experimental evidence that the dynamical 'glass transition' transition directly depends on the dynamics of the solvent. For example, the neutron scattering measurements by Tsai et al.

[20] have shown that this type of dynamical transition, which often directly reflects itself in the cessation of protein biological activity[21], is not observed in dried lysozyme over a large temperature range between 40 K and 500 K and that both this dynamical transition and the protein denaturation temperature can be tuned by varying the amount of water. Pacciaroni et al.[22] later showed that the dynamical transition in glycerol-water solutions of lysozyme can be tuned by varying the water content. This solvent-protein coupling phenomenon has been characterized as “slaving” and has been discussed in numerous previous computational and experimental studies covering a range of proteins and solvents, as nicely reviewed by Dirama et al.[14-18] [16-18]Schiro et al.[23] have recently shown that this type of coupling arises for both a globular (maltose binding protein) and the intrinsically disordered protein (htau40). Given that protein dynamics is so greatly influenced by the solvent, it is crucial to describe the solvent dynamics faithfully if we want to realistically simulate the dynamics of proteins.

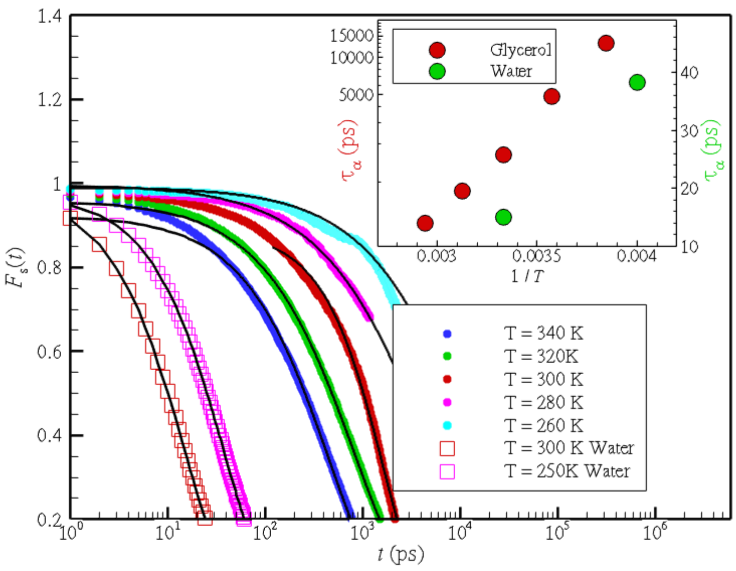


**Figure S.2** The self-intermediate scattering function $F_s(q,t)$, the Fourier transform of $G_s(r, \Delta t)$ at designated temperatures based on CHARM36 potentials for water and glycerol. The inset shows the structural or "alpha" relaxation time $\tau_\alpha$ deduced from the decay of $F_s(q^*,t)$ where $q^*$ is taken to have a value associated with the peak in the static structure, a scale that defines the average inter-particle distance. $F_s(q^*,t)$ has been normalized by the static structure factor so that $F_s(q^*,0)$ approaches 1 as $t$ approaches $t$ = 0. The decay of $F_s(q^*,t)$ for water is almost perfectly exponential, while the decay of $F_s(q^*,t)$ for glycerol is well described by a stretched exponential, $F_s(q^*,t) = \exp(-(t/\tau_\alpha)^\beta)$ with a stretching exponent $\beta$ near 0.8 for all the $T$ simulated. This stretching is a characteristic feature of glass-forming liquids [24, 25] so we have additional evidence of the dynamic homogeneity of TIP3P water. Note that the rapid growth of $\tau_\alpha$ upon cooling makes equilibrium simulations difficult since computational times must then be significantly longer than this time.

## B. Average amplitude of local atomic motion $\langle u^2 \rangle$ within ubiquitin at room temperature

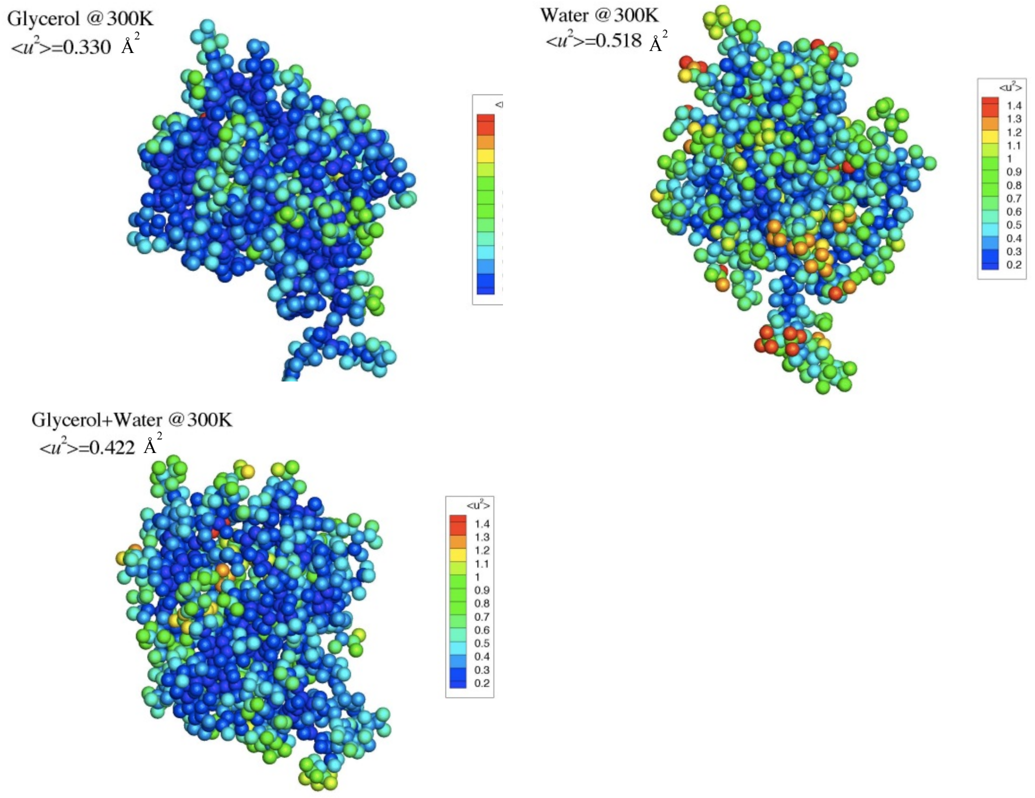


**Figure S.3.** Influence of the addition of water on the average amplitude of local atomic motion $\langle u^2 \rangle$ within ubiquitin at room temperature. The changes occur non-uniformly; the enhancement of molecular motion is greatest near the protein's core and the protein-water boundary. More specifically, the relatively rigid alpha helix and beta sheet structures are positioned around the mobile core of the protein. This effect is illustrated in an averaged way in Figure 2(b) of the text where $\langle u^2 \rangle$ has been radially averaged for the protein. Consistent with our simulation findings, Lindorff-Larsen[26] have observed a high mobility in the core of ubiquitin on a *ps* timescale and suggest that this is a common property of many proteins.

## References


1. Price DJ, Brooks CL. A modified TIP3P water potential for simulation with Ewald summation. *Journal of Chemical Physics* **121**, 10096-10103 (2004).

2. Mark P, Nilsson L. Structure and dynamics of the TIP3P, SPC, and SPC/E water models at 298 K. *Journal of Physical Chemistry A* **105**, 9954-9960 (2001).

3. Yeh IC, Hummer G. System-size dependence of diffusion coefficients and viscosities from molecular dynamics simulations with periodic boundary conditions. *J Phys Chem B* **108**, 15873-15879 (2004).

4. Zhang Y, Tyagi M, Mamontov E, Chen SH. Quasi-elastic neutron scattering studies of the slow dynamics of supercooled and glassy aspirin. *Journal of Physics-Condensed Matter* **24**, 064112 (2012).

5. Starr FW, Sciortino F, Stanley HE. Dynamics of simulated water under pressure. *Phys Rev E* **60**, 6757-6768 (1999).

6. Starr FW, Douglas JF, Sastry S. The relationship of dynamical heterogeneity to the Adam-Gibbs and random first-order transition theories of glass formation. *Journal of Chemical Physics* **138**, 12A541 (2013).

7. Jahn DA, Akinkunmi FO, Giovambattista N. Effects of Temperature on the Properties of Glycerol: A Computer Simulation Study of Five Different Force Fields. *J Phys Chem B* **118**, 11284-11294 (2014).

8. Angell CA. Formation of Glasses from Liquids and Biopolymers. *Science* **267**, 1924-1935 (1995).

9. Betancourt BAP, Hanakata PZ, Starr FW, Douglas JF. Quantitative relations between cooperative motion, emergent elasticity, and free volume in model glass-forming polymer materials. *P Natl Acad Sci USA* **112**, 2966-2971 (2015).

10. Zondervan R, Kulzer F, Berkhout GCG, Orrit M. Local viscosity of supercooled glycerol near T-g probed by rotational diffusion of ensembles and single dye molecules. *P Natl Acad Sci USA* **104**, 12628-12633 (2007).

11. Chen B, Sigmund EE, Halperin WP. Stokes-Einstein relation in supercooled aqueous solutions of glycerol. *Phys Rev Lett* **96**, 145502 (2006).

12. Phillips JC*, et al.* Scalable molecular dynamics with NAMD. *J Comput Chem* **26**, 1781-1802 (2005).

13. Merling WL, Mileski JB, Douglas JF, Simmons DS. The Glass Transition of a Single Macromolecule. *Macromolecules* **in press**, (2016).

14. Dirama TE, Curtis JE, Carri GA, Sokolov AP. Coupling between lysozyme and trehalose dynamics: Microscopic insights from molecular-dynamics simulations. *Journal of Chemical Physics* **124**, 034901 (2006).

15. Dirama TE, Carri GA, Sokolov AP. Coupling between lysozyme and glycerol dynamics: Microscopic insights from molecular-dynamics simulations. *Journal of Chemical Physics* **122**, 244910 (2005).

16. Dudowicz J, Freed KF, Douglas JF. Solvation of polymers as mutual association. I. General theory. *Journal of Chemical Physics* **138**, 164901 (2013).

17. Dudowicz J, Freed KF, Douglas JF. Solvation of polymers as mutual association. II. Basic thermodynamic properties. *Journal of Chemical Physics* **138**, 164902 (2013).

18. Dudowicz J, Freed KF, Douglas JF. Phase behavior and second osmotic virial coefficient for competitive polymer solvation in mixed solvent solutions. *Journal of Chemical Physics* **143**, 194901 (2015).

19. Dudowicz J, Freed KF, Douglas JF. Generalized Entropy Theory of Polymer Glass Formation. *Advances in Chemical Physics, Vol 137* **137**, 125-222 (2008).

20. Tsai AM, Neumann DA, Bell LN. Molecular dynamics of solid-state lysozyme as affected by glycerol and water: A neutron scattering study. *Biophys J* **79**, 2728-2732 (2000).

21. Roh JH, Novikov VN, Gregory RB, Curtis JE, Chowdhuri Z, Sokolov AP. Onsets of anharmonicity in protein dynamics. *Phys Rev Lett* **95**, 038101 (2005).

22. Paciaroni A, Cinelli S, Onori G. Effect of the environment on the protein dynamical transition: A neutron scattering study. *Biophys J* **83**, 1157-1164 (2002).

23. Schiro G*, et al.* Translational diffusion of hydration water correlates with functional motions in folded and intrinsically disordered proteins. *Nat Commun* **6**, 6490 (2015).

24. Bohmer R, Ngai KL, Angell CA, Plazek DJ. Nonexponential Relaxations in Strong and Fragile Glass Formers. *Journal of Chemical Physics* **99**, 4201-4209 (1993).

25. Zhang H*, et al.* Role of string-like collective atomic motion on diffusion and structural relaxation in glass forming Cu-Zr alloys. *Journal of Chemical Physics* **142**, 164506 (2015).

26. Lindorff-Larsen K, Best RB, DePristo MA, Dobson CM, Vendruscolo M. Simultaneous determination of protein structure and dynamics. *Nature* **433**, 128-132 (2005).
